# Pericardiocentesis: ultrasound guidance is essential

**DOI:** 10.1186/s13089-022-00259-5

**Published:** 2022-02-14

**Authors:** Pablo Blanco, Liliana Figueroa, María Fernanda Menéndez, Belén Berrueta

**Affiliations:** 1 High-Dependency Unit (UCIM), Hospital “Dr. Emilio Ferreyra”, 4801, 59 Ave., 7630 Necochea, Argentina; 2 Department of Teaching and Research, Hospital “Dr. Emilio Ferreyra”, 4801, 59 Ave., 7630 Necochea, Argentina

**Keywords:** Pericardial effusion, Point-of-care, Ultrasonography, Pericardiocentesis

## Abstract

**Background:**

Pericardial effusion is a common entity which may have important implications in patient’s prognosis. In several cases, pericardiocentesis is indicated for diagnostic and/or therapeutic purposes.

**Case presentation:**

A blind pericardiocentesis failed in a 95-year-old woman admitted to the emergency department with a large pericardial effusion incidentally diagnosed in the ambulatory setting. Ultrasound-guided pericardiocentesis aided in easily accessing to the pericardial cavity, without periprocedural complications.

**Conclusions:**

Ultrasound-guided pericardiocentesis is simple, safe and effective, and should replace the blind technique. This procedure should be part of the armamentarium of ultrasound-guided practices of emergency or critical care physicians.

**Supplementary Information:**

The online version contains supplementary material available at 10.1186/s13089-022-00259-5.

## Background

Pericardial effusion (i.e., accumulation of fluid between the two layers of pericardium) may occur for several causes including congestive heart failure or pulmonary hypertension, cancer, infections, iatrogenic, metabolic, trauma or connective tissue diseases; however, up to 50% remain idiopathic [1, 2]. Pericardial effusions (even large ones) may be found incidentally on chest X-rays or echocardiograms performed for other reasons [1].

Point-of-care transthoracic echocardiography aids in the diagnosis of pericardial effusion, determining its size and distribution, assessing hemodynamic repercussion and also in guiding pericardiocentesis if indicated.

Regarding the echocardiographic assessment, it is of paramount relevance that physicians bear in mind that a pericardial effusion, although circumferential, may not be always uniformly distributed around the heart. Therefore, multiple views should be performed to best estimate the size of the effusion, the site of maximal fluid accumulation and therefore, the best pericardicentesis route, if indicated.

We report the case of a patient presenting with a large pericardial effusion in whom a blind pericardiocentesis failed and how ultrasound guidance aided in performing pericardiocentesis in an easy and safe manner, improving patient care.

## Case presentation

A 95-year-old woman was admitted to the emergency department after being incidentally diagnosed of a large pericardial effusion in an echocardiogram performed by the cardiologist in the ambulatory setting. Relevant medical history revealed the implantation of a dual-chamber pacemaker 4 months before. Vital signs showed a heart rate of 70 beats per minute, respiratory rate of 16 breaths per minute, blood pressure of 130/80 mmHg, pulse oximetry of 96% breathing room air and axillary temperature of 36 °C (96.8 °F). Physical examination showed a lucid, comfortable and collaborative patient. She was asymptomatic for dyspnea or chest pain. Respiratory examination showed a reduced vesicular breath sound in the basal aspect of both hemithorax. Cardiac examination showed muffled heart sounds, no jugular vein distention, minor edema in both legs and no signs of hypoperfusion. The rest of the physical examination was unremarkable. Chest X-ray showed a “water-bottle” cardiac silhouette and interstitial infiltrates in the right lung; blunted costophrenic angles were noted on both sides. Dual-chamber pacemaker generator was seen in the left side below the clavicle, with atrial and ventricular leads placed in an adequate position. Surface electrocardiogram depicted atrial and ventricular pacing spikes with appropriate sensing and capture; QRS complexes were of normal amplitude (left bundle-branch block morphology). Blood sample analysis was entirely normal. While an ultrasound machine was available in the emergency department, the attending physician (without skills in point-of-care ultrasound), based on the echocardiogram report, decided to perform a blind diagnostic pericardiocentesis via the subxiphoid approach. After failed four attempts (“dry taps”), the procedure was stopped and the patient was derived to the general ward for cardiac consultation. The next day, the patient was transferred to the high-dependency unit (HDU) for monitoring and to continue the diagnostic workup of the pericardial effusion.

**Fig. 1:**
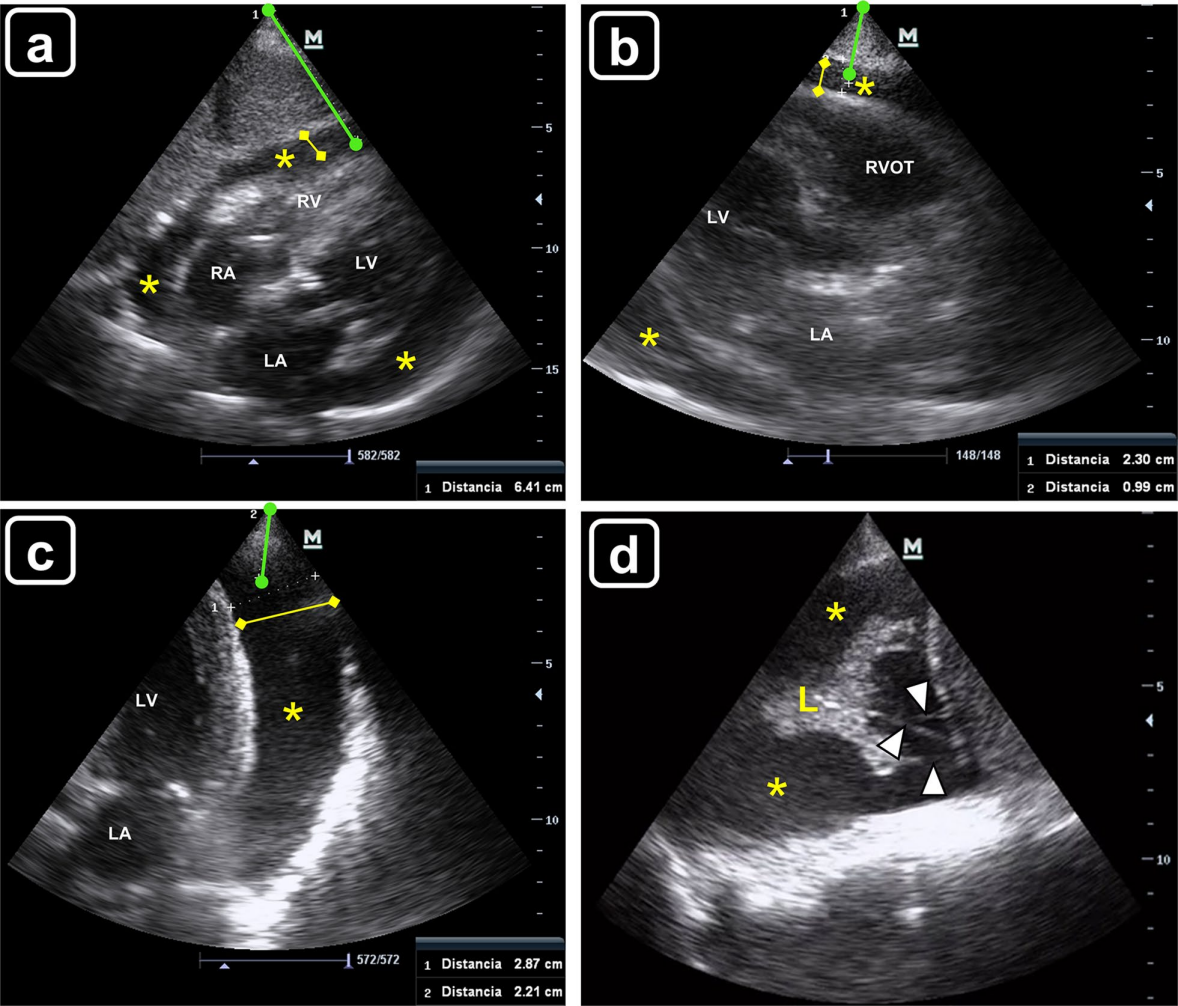
Point-of-care echocardiogram showing the circumferential pericardial effusion (asterisks), the pericardial fluid thickness (yellow line) and the distance to reach the pericardial cavity from the skin (green line); **a** subcostal four chamber view, **b** parasternal long axis view, **c** apical four chamber view, *RA* right atrium; *RV* right ventricle; *LA* left atrium; *LV* left ventricle; *RVOT* right ventricular outflow tract. **d** Left-sided pleural effusion (asterisks) with fibrin strands (arrowheads); *L* lung consolidation

**Fig. 2:**
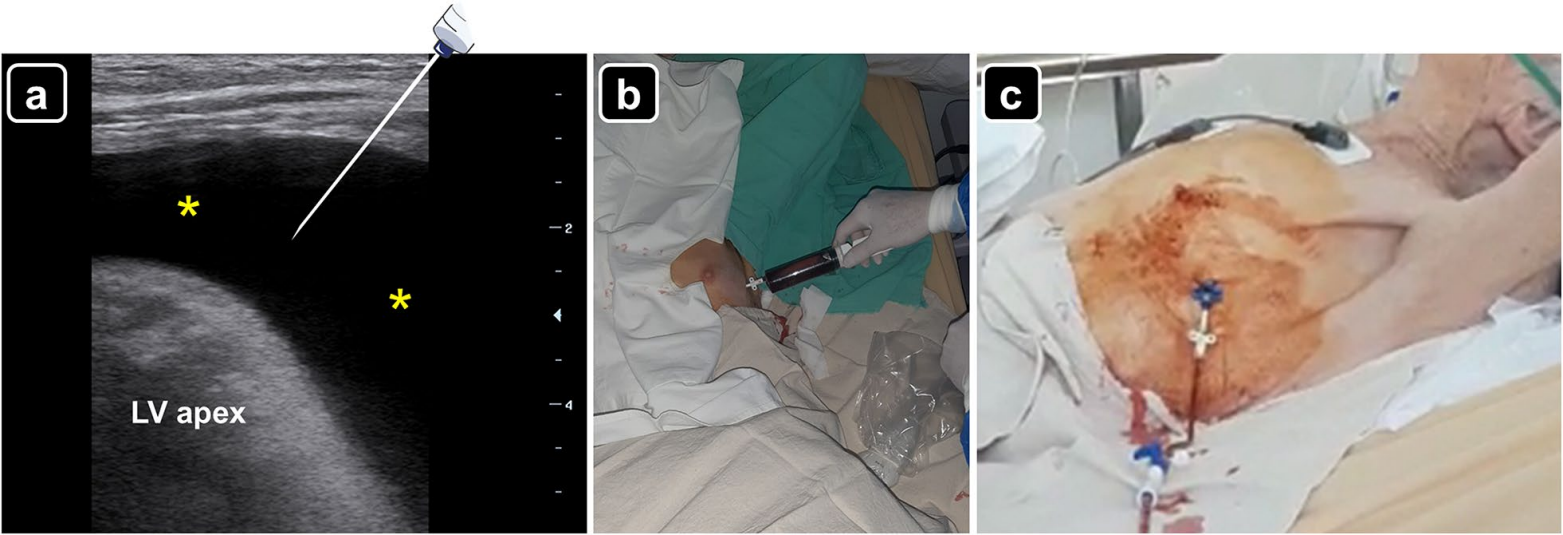
**a** Pericardial effusion (asterisks) observed with a linear probe in the cardiac apex region. A needle-syringe is illustrating the path to reach the pericardial cavity. *LV* left ventricular apex. **b** Pericardiocentesis via the apical approach freely obtaining an abundant hematic fluid. **c** Pericardial catheter drainage inserted through the Seldinger technique

On patient’s admission to the HDU, clinical status did not show changes compared with the previous day. A point-of-care echocardiogram showed a large circumferential pericardial effusion with an asymmetric distribution, being larger along the lateral wall of the left ventricle with the patient supine (Table 1 and Fig. 1a–c). Systolic function of both ventricles was normal. No diastolic ventricular collapse was noted; pacing leads were in the correct position (one in the right atrium; one in the right ventricle). Lung ultrasound showed a moderate pleural effusion with fibrin strands was noted on the left hemithorax (Fig. 1d), while a mild pleural effusion was also depicted on the right side. To aid in etiological diagnosis and given that no clinical signs of cardiac tamponade were present, a left-sided thoracocentesis was performed, draining 500 ml of a citrine pleural fluid with transudate features on chemical analyses; microbiological study was normal while no neoplastic cells were detected on pleural fluid cytology. The next day, given the etiologic uncertainness, an ultrasound-guided pericardiocentesis under local anesthesia was performed via the apical approach. To best do that, given the thin thoracic wall of the patient, a linear probe was used to best define the site of insertion and the path of the needle, while avoiding the pleura (Fig. 2a and Additional file 1: Video S1). A single needle puncture under real-time ultrasound guidance allowed to easily enter the pericardial cavity, freely obtaining a hematic fluid (Fig. 2b). With the Seldinger technique, a 14G-30 cm in length catheter was placed in the pericardial cavity (Fig. 2c). No periprocedural complications were detected. A total of 1300 ml was drained in the first day. After confirming that the pericardial effusion was completely evacuated by serial point-of-care echocardiogram, the catheter was retired on day 3. Cytological analysis of the pericardial fluid did not reveal neoplastic cells, while microbiological study was negative as well.

**Table 1:** Pericardial fluid thickness and distance to reach the pericardial cavity for the three pericardiocentesis approaches of the patient

Pericardiocentesis approach	Pericardial fluid thickness (mm)	Approximate distance to enter in the pericardial cavity (mm)
Subxiphoid	13.6	64
Left parasternal	10	23
Apical	29	22

A benign pericardial effusion related to pacemaker implantation was the most plausible explanation and the patient was sent back to the general ward to further care.

## Discussion

Pericardiocentesis is clearly indicated in tamponade; in patients without hemodynamic compromise, pericardiocentesis is indicated for symptomatic moderate to large effusion non-responsive to medical therapy, when tuberculous, bacterial or neoplastic pericarditis is suspected, or in case of chronic (lasting more than 3 months) large pericardial effusion (> 20 mm on echocardiography in diastole) [1–3].

Other options to manage the pericardial effusion include surgical techniques, such as subxiphoid pericardiotomy and partial or complete pericardiectomy, providing low rates of fluid reaccumulation. However, surgical approaches were associated with high mortality and morbidity. Indeed, the 30-day mortality rates of surgical techniques ranged from 20 to 50% [4].

There are no absolute contraindications to pericardiocentesis when cardiac tamponade occurs. However, in tamponade related to aortic dissection and post-infarction rupture of the free wall (surgical tamponade), pericardiocentesis carries the risk of aggravating the dissection or myocardial rupture via rapid pericardial decompression and restoration of systemic arterial pressure. In these cases, if surgical management is not immediately available, or if the patient is too unstable, pericardiocentesis and drainage of small amounts of the hemopericardium can be attempted in order to maintain hemodynamic stability as a bridge to emergency surgery. Relative contraindications include uncorrected coagulopathy, anticoagulant therapy or thrombocytopenia (platelet count  < 50,000/mm^3^) [2, 3].

Due to the proximity of several vital structures, including the heart itself, diagnostic and therapeutic pericardial effusion drainage should be performed with ultrasound guidance, which has a reported procedural success rate that approaches 97% [4]. Unfortunately, in many countries around the globe, such as Argentina, because of the lack of expertise in point-of-care ultrasound, blind subxiphoid pericardiocentesis is the first approach that is still performed in many centers. This practice carries with a morbidity rate that approaches 20% and mortality rates as high as 6% [4]. In contrast, for practitioners proficient in echo-guided pericardiocentesis, the rate of major complications is 0.3–3.9%, and the rate of minor complications is 0.4–20% [3]. Given the data mentioned above, ultrasound guidance is of key relevance before, during, and after pericardiocentesis [2].

**Fig. 3:**
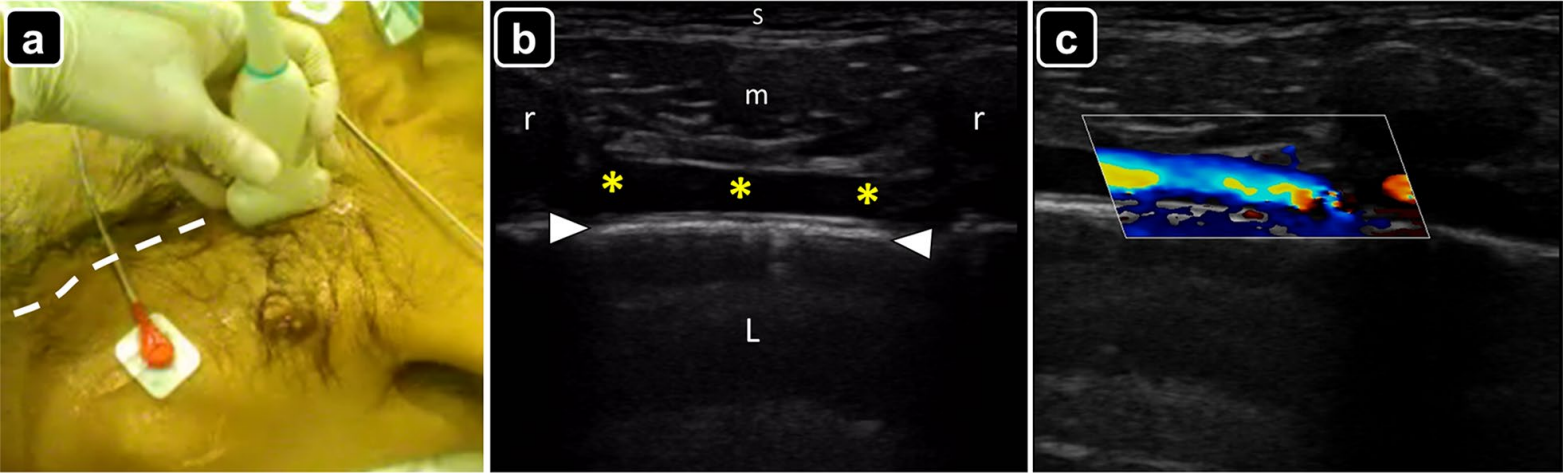
**a** Technique for localization of the internal thoracic vessels (dotted line) using a linear probe along the left parasternal line. **b** Identification of the internal thoracic vessels (asterisks) on ultrasound; *s* skin and subcutaneous tissue; *r* ribs; *arrowheads* pleural line; *L* lung. **c** Color Doppler showing blood flow in the internal thoracic vessels

There are three approaches to perform ultrasound-guided pericardiocentesis: subcostal, left parasternal and apical. The preferred route is often the thoracic wall (intercostal) over the subcostal approach [3–7], given that in the former there is a shorter distance for the needle to reach the pericardial cavity. The phased-array probe is often used to guide the procedure; however, a convex probe may also be used, particularly in thin patients. A linear probe may aid in defining the pleura and internal thoracic vessels and excluding them from the needle track (Fig. 3) [6].

Ultrasound-guided pericardiocentesis can be carried out by one or two operators. In the former, the operator performs the ultrasound scan and the puncture and drainage; in the latter, one operator performs the ultrasound scan and another the puncture and drainage. To the authors’ knowledge, there is no formal recommendation in the literature regarding this point; therefore, it is left to the free choice of the center.

In our HDU as in other centers, we perform pericardiocentesis using a central venous cannulation set containing a needle of 18 G × 7 cm in length, a guidewire of 0.032 in. × 60 cm in length, a dilator and a catheter of 14 G × 30 cm in length. The catheter is placed in the pericardial cavity with the modified Seldinger technique. The entire procedure is performed under normal sterile barrier precautions.

The point of needle entry should be wisely selected based on the site where the effusion accumulation is maximal and closest to the transducer, while avoiding key structures, such as the liver, myocardium, pleura or internal thoracic vessels [7]. When using intercostal approaches (i.e., parasternal or apical), the needle should be inserted along the superior border of the rib as a way to prevent inadvertent injury to the intercostal vessels, which runs along the inferior rib border. A sterile needle guide can be used, if available. As advanced, the needle is observed in real-time entering in the pericardial cavity; after aspiration a few milliliters of fluid, 5 ml of agitated saline are injected through the needle, aiding in confirming that it is in the pericardial cavity and not in a cardiac chamber [8] (Additional files 2, 3: Videos S2, S3). After this, the catheter is finally placed via the Seldinger technique.

The advantages and disadvantages of each pericardiocentesis approach are summarized in Table 2.

**Table 2:** Advantages and disadvantages of pericardiocentesis approaches (modified from [7])

Pericardiocentesis approach	Advantages	Disadvantages
Subxiphoid	Extrapleural route	Liver injury Colon or stomach perforation Long distance to reach the pericardial cavity Irritation to diaphragm or phrenic nerve
Left parasternal	Direct route to reach the pericardial cavity	Internal thoracic vessels injury Pneumothorax
Apical	Direct route to reach the pericardial cavity	Pneumothorax Ventricular apex piercing (ventricular arrhythmias)

The most serious complications of pericardiocentesis include death, injury of the cardiac chambers, laceration of the coronary arteries, intercostal or internal thoracic vessels injury, puncture of the abdominal viscera or peritoneal cavity, pneumothorax, pneumopericardium, ventricular arrhythmias and pericardial decompression syndrome [3–7]. Minor complications include transient vasovagal hypotension and bradycardia, supraventricular arrhythmias and pleuropericardial fistulas [3].

Regarding training in echo-guided pericardiocentesis, there are several simulation models (even at a very low cost) that allow learners to gain confidence with the procedure without posing any patient at risk [9]. To authors’ knowledge, a minimum of ultrasound-guided pericardiocentesis procedures is not established in the core curriculum of point-of-care ultrasound training for emergency or critical care physicians; however, similar to vascular cannulation, achieving at least 10 fully supervised procedures seems rational.

## Conclusions

In patients in whom pericardiocentesis is indicated, ultrasound-guided pericardiocentesis should be always performed considering its best safety profile and high effectiveness, compared with the blind approach. As such, this procedure must be included in the core curriculum of point-of-care ultrasound training for emergency and critical care physicians.

## Supplementary Information

**Additional file 1: Video S1.** Pericardial effusion observed with a linear probe in the cardiac apex region, excluding the pleura from the needle track.

**Additional file 2: Video S2.** Technique for preparation of the agitated saline solution.

**Additional file 3: Video S3.** Injection of agitated saline solution for confirming that the needle is in the pericardial cavity instead of a cardiac chamber.

## Data Availability

Not applicable.
